# Biostimulatory and Inhibitory Effects of Natural Extracts on *Vigna radiata*: Concentration-Dependent Responses

**DOI:** 10.3390/molecules31122030

**Published:** 2026-06-10

**Authors:** Barbara Drygaś, Joanna Kreczko, Tomasz Piechowiak, Marta Jańczak-Pieniążek, Czesław Puchalski, Ireneusz Kapusta, Ewa Szpunar-Krok

**Affiliations:** 1 Department of Bioenergetics, Food Analysis and Microbiology, University of Rzeszow, 35-601 Rzeszów, Poland; 2 Utrica Technologies Sp. z o.o., Stanisława Lema St. 4A/1, 80-126 Gdańsk, Poland; 3 Department of Chemistry and Food Toxicology, University of Rzeszow, 35-601 Rzeszów, Poland; 4 Department of Crop Production, University of Rzeszow, 35-601 Rzeszów, Poland; mjanczak@ur.edu.pl (M.J.-P.); eszpunar@ur.edu.pl (E.S.-K.); 5 Department of Food Technology and Human Nutrition, University of Rzeszow, 35-601 Rzeszow, Poland; ikapusta@ur.edu.pl

**Keywords:** biostimulants, algae extracts, plant extracts, mung bean, chlorophyll content, chlorophyll fluorescence

## Abstract

Natural extracts derived from plants and algae are increasingly recognized for their ability to modulate plant growth and physiological processes due to their rich content of bioactive compounds. Depending on their composition and concentration, these extracts may act as biostimulants, enhancing germination, stress tolerance, and antioxidant activity, or may exhibit inhibitory effects. This study aimed to determine the effects of extracts from *Ascophyllum nodosum*, *Fucus vesiculosus*, and *Sideritis scardica*, prepared using different solvents, on germination parameters, early growth, chlorophyll content, chlorophyll a fluorescence parameters, secondary metabolite levels (phenolic compounds and flavonoids), and antioxidant activity in mung bean (*Vigna radiata* L.). The results showed that aqueous and hydroethanolic extracts were generally safe for germination, maintaining high germination capacity, whereas 10% ethanolic extracts exhibited pronounced inhibitory effects, reducing germination energy to as low as 8%. Secondary metabolism was markedly affected, particularly by *S. scardica* extracts, with dose-dependent changes observed in total phenolic and flavonoid contents. Despite these biochemical alterations, chlorophyll fluorescence parameters remained stable, indicating the absence of photoinhibitory stress. Overall, the results indicate that the biological activity of the extracts depends strongly on solvent type and concentration, which determine whether they act as biostimulants or inhibitors by modulating early growth, metabolism, and physiological status. These findings provide a basis for the development of optimized plant- and algae-based biostimulant formulations.

## 1. Introduction

Mung bean (*Vigna radiata* L.) is a valuable crop with high nutritional potential, and its microgreens are a rich source of vitamins, phenolic compounds, and flavonoids [1,2,3,4]. Previous studies have shown that germination enhances the accumulation of numerous health-promoting bioactive compounds, including polyphenolic compounds, flavonoids, vitamins C and E, and overall antioxidant activity [5]. These compounds are widely recognized for their antioxidant, antidiabetic, antimicrobial, antihyperlipidemic, antihypertensive, anti-inflammatory, anticancer, antitumor, and antimutagenic properties [5]. Mung beans are also valued for their high protein content, which accounts for approximately 20–25% of seed dry weight. Their consumption has steadily increased, particularly in combination with cereal products [6,7]. The protein fraction of mung beans is particularly rich in essential amino acids such as phenylalanine, leucine, isoleucine, valine, tryptophan, arginine, methionine, and lysine [8]. Numerous secondary metabolites present in mung beans have been identified as beneficial to human health, including flavonoids (such as flavones, isoflavones, and isoflavonoids), phenolic acids (e.g., gallic, vanillic, caffeic, cinnamic, protocatechuic, shikimic, and p-hydroxybenzoic acids), and other organic acids [9]. The nutritional value and health-promoting properties of mung beans are further enhanced during germination [10]. Previous studies indicate that germination significantly improves the levels of various nutrients and metabolites in mung beans. For instance, sprouting reduces triacylglycerol content; increases concentrations of free amino acids and total phenolic acids; and modifies the composition of fatty acid methyl esters, free fatty acids, monosaccharides, and disaccharides [11]. In addition, sprouts contain higher levels of antioxidant compounds, particularly polyphenolic compounds, compared with ungerminated seeds [5,11]. Consequently, germination is considered an effective approach for improving both the nutritional and medicinal qualities of mung beans [5].

Plant- and algae-derived biostimulants are becoming increasingly important in agriculture due to their potential to improve seed emergence, seedling growth, and photosynthetic efficiency while reducing the reliance on synthetic agrochemicals. Bioactive compounds found in extracts can modulate plant physiological processes, including germination, photosynthesis, stress tolerance, and resistance to environmental stresses [12,13,14]. Extracts from brown algae such as *Ascophyllum nodosum* and *Fucus vesiculosus* have been extensively studied and often demonstrate biostimulant effects [15,16,17,18,19,20,21,22,23,24]. However, there is limited research on *Sideritis scardica* as a plant biostimulant; most of the literature focuses on its pharmaceutical, antioxidant, or anti-inflammatory properties or its chemical composition [25,26,27].

This study examined the effects of plant- and algae-derived extracts on the germination and early growth of *Vigna radiata* L. microgreens. The primary objective was to evaluate the effects of selected natural extracts on plants during the early stages of development and to explore their potential application in microgreen cultivation. It was hypothesized that these natural extracts would enhance plant growth parameters, increase antioxidant activity, and improve chlorophyll content and related physiological traits. To test this hypothesis, several parameters were assessed, including germination energy and capacity, the Maguire and Pieper indices, plant growth and biomass, relative chlorophyll content, chlorophyll a fluorescence parameters, total phenolic content (TPC), total flavonoid content (TFC), and antioxidant activity.

## 2. Results

### 2.1. Analysis of the Extracts—Determination of Polyphenolic Compounds Profile

Table 1 presents the phenolic compounds identified in the *Sideritis scardica* extract using UPLC-PDA-MS/MS. A total of 20 phenolic compounds were identified in the extract, belonging to three main groups: (I) phenolic acids (e.g., 3-O-caffeoylquinic acid), (II) phenylethanoid glycosides (including echinacoside, verbascoside, forsythoside, and leucoseptoside), and (III) O-glycosylated flavones (derivatives of hypolaetin and isoscutellarein, including acetylated and methylated forms).

Table 2 presents the quantitative contents of the chemical compounds listed in Table 1. It illustrates how the solvent used in the extraction process affects the recovery of individual chemical compounds.

Phenolic compounds present in the analyzed brown algae were also determined (Table 3) and their quantitative analysis was presented in Table 4. UPLC-PDA-MS/MS analysis of the algal extracts (Table 3 and Table 4) revealed the presence of four phlorotannins—phloroglucinol, phlorethol, fucophloretol, and fuhalol. These compounds are characteristic polyphenolic compounds of brown algae that belong to the phloroglucinol oligomer family. *Fucus vesiculosus* extracts had the highest total phlorotannin concentration (8.28 µg/mL), while *Ascophyllum nodosum* extracts had a significantly lower concentration (2.76 µg/mL). Phloroglucinol was the dominant compound in both cases, reaching 2.99 µg∙mL^−1^ in *F. vesiculosus* extract and 1.10 µg∙mL^−1^ in *A. nodosum* extract. The phlorotannin profile differed between the two algal species. Fucophloretol was identified only in the *F. vesiculosus* extract and was not detected in the *A. nodosum* extract. Fuhalol, on the other hand, was present at similar concentrations in both materials (approximately 0.40 µg∙mL^−1^). Importantly, all identified phlorotannins were detected exclusively in aqueous extracts and not in hydroethanolic or ethanolic extracts. This suggests that the type of solvent used significantly impacts the extraction of these compounds and that their greater water solubility favors isolation in aqueous extracts.

### 2.2. Analysis of Germination Energy and Capacity

The analysis of mung bean germination energy and capacity revealed clear differences depending on the type of extract used. The control and most aqueous (W) and hydroethanolic (WE) extracts maintained very high germination energy and capacity (96–100%). In contrast, ethanolic extracts (E) at a high concentration of 10% significantly reduced germination parameters. For the 10% ethanolic extract of *Ascophyllum nodosum* (ASC), germination energy was 24%, and germination capacity after 7 and 12 days was only 57.3%. A similar effect was observed for *Fucus vesiculosus* (FUC) E 10% (energy 40%, germination capacity ~60%) and *Sideritis scardica* (SID) E 10% (energy 8%, germination capacity 42.7–46.7%). A slight decrease in germination energy and capacity was also observed for the 10% hydroethanolic extract of *A. nodosum*. A simplified summary of the main concentration-dependent effects of the tested extracts on mung bean germination is presented in Table 5. The complete dataset, including all treatment variants, germination capacity after 12 days, and detailed statistical analysis, is provided in Supplementary Table S1.

At lower concentrations (1%, 0.1%, and 0.01%), the ethanolic extracts did not differ significantly from the control, maintaining germination parameters at 98–100%. This indicates that the inhibitory effect associated with ethanolic extracts became apparent only at the highest concentration. In contrast, aqueous and hydroethanolic extracts did not cause any reduction in germination energy or capacity, regardless of the dose.

In summary, aqueous and hydroethanolic extracts were safe for mung bean germination, whereas 10% ethanolic extracts exhibited inhibitory effects, reducing both germination rate and final germination capacity. These results demonstrate that the choice of solvent and its concentration are critical factors influencing the biostimulatory properties of the extracts.

**Figure 1:**
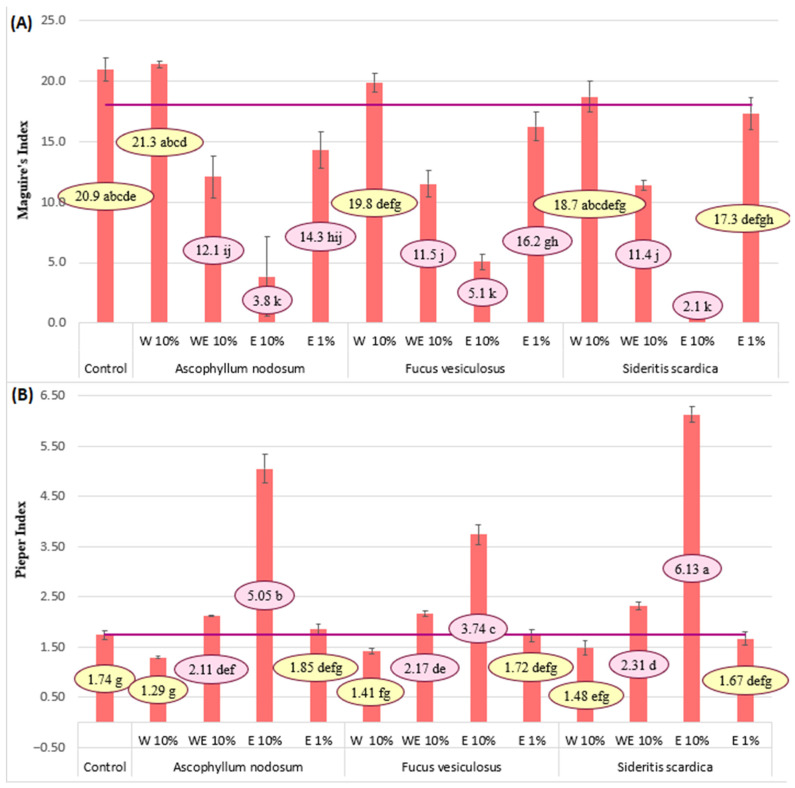
Selected parameters describing mung bean germination as influenced by extract type and extract concentration. (**A**) Maguire’s Index; (**B**) Pieper Index. The horizontal line indicates the mean value calculated for the complete dataset presented in Supplementary Figure S1. W—aqueous extract; WE—hydroethanolic extract; E—ethanolic extract. Values are presented as mean ± SD. Different letters indicate significant differences between mean values according to Tukey’s test (*p* < 0.05). Values highlighted in yellow indicate treatments not significantly different from the control whereas pink labels indicate treatments significantly different from the control.

Figure 1 presents the principal concentration-dependent trends observed for the germination indices of mung bean seeds treated with selected extract variants. The simplified figure highlights the contrasting responses observed between low- and high-concentration treatments and improves the clarity of the main treatment effects. The complete dataset including all concentrations and treatment variants is provided in Supplementary Figure S1.

Figure S1 presents the Maguire index for mung bean seeds germinated on filter papers moistened with different extracts. Most values were close to the mean; however, significant decreases were observed for the concentrated 10% ethanolic extracts of *A. nodosum*, *F. vesiculosus*, and *S. scardica*. A negative effect on the Maguire index was also noted for 10% hydroethanolic extracts. Additionally, a notable reduction in the Maguire index was observed for seeds treated with 1% ethanolic extracts from the brown algae. Figure S1 also presents the Pieper index for the germinating seeds. The mean value was approximately 1.8, with most values ranging between 1.2 and 2.0. However, markedly higher values were observed for 10% ethanolic extracts: *Ascophyllum nodosum* (5.05), *Fucus vesiculosus* (3.74), and *Sideritis scardica* (6.13). These results indicate that high-concentration ethanolic extracts negatively affect the germination indices of mung bean seeds.

### 2.3. Seedling Length and Fresh Weight

Table 6 presents the average growth and fresh weight of mung bean microgreens for different experimental variants.

In general, aqueous and hydroethanolic extracts at low concentrations did not negatively affect microgreens growth or fresh weight. Negative effects on these parameters were observed in plants treated with 10% hydroethanolic extracts of *A. nodosum*, *F. vesiculosus*, and *S. scardica*. A tendency toward increased microgreens height and weight was also observed after the application of aqueous and hydroethanolic extracts (except at the 10% concentration) of *S. scardica*.

### 2.4. Chlorophyll Content in Young Leaves

**Figure 2:**
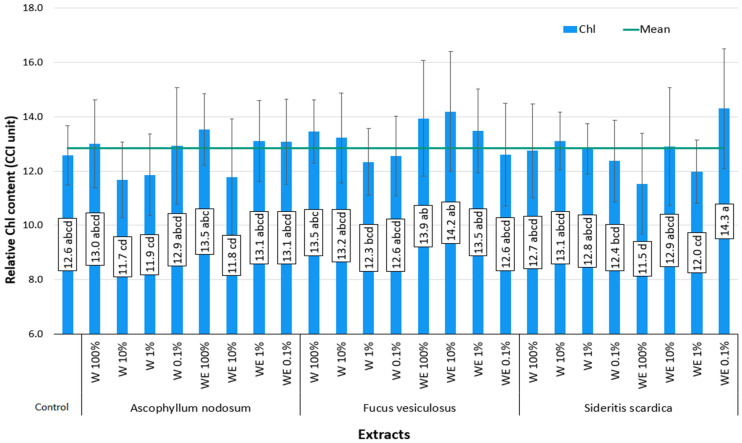
Relative chlorophyll content in mung bean microgreens as a function of the type and extract concentration. W—water (aqueous) extract; WE—hydroethanolic extract. Mean values ± SD. Means with the same letters do not differ significantly at *p* < 0.05 in Tukey’s test.

The applied extracts did not significantly affect chlorophyll content in young mung bean leaves (Figure 2).

There was a tendency toward higher relative chlorophyll content in plants treated with, among others, hydroethanolic extracts of *F. vesiculosus* at concentrations of 100% and 10%, as well as the hydroethanolic extract of *S. scardica* at 0.1%. Slightly lower values compared with the control were observed in plants irrigated with 10% and 1% aqueous extracts of *A. nodosum*, the 1% hydroethanolic extract of the same alga, and a 100% hydroethanolic extract of *S. scardica*. Although these differences were not statistically significant relative to the control, statistically significant differences were found among the hydroethanolic *S. scardica* extracts regarding their effects on total chlorophyll content. The lowest concentration increased chlorophyll content, whereas the highest concentration reduced this parameter.

### 2.5. Chlorophyll Fluorescence Parameters

**Figure 3:**
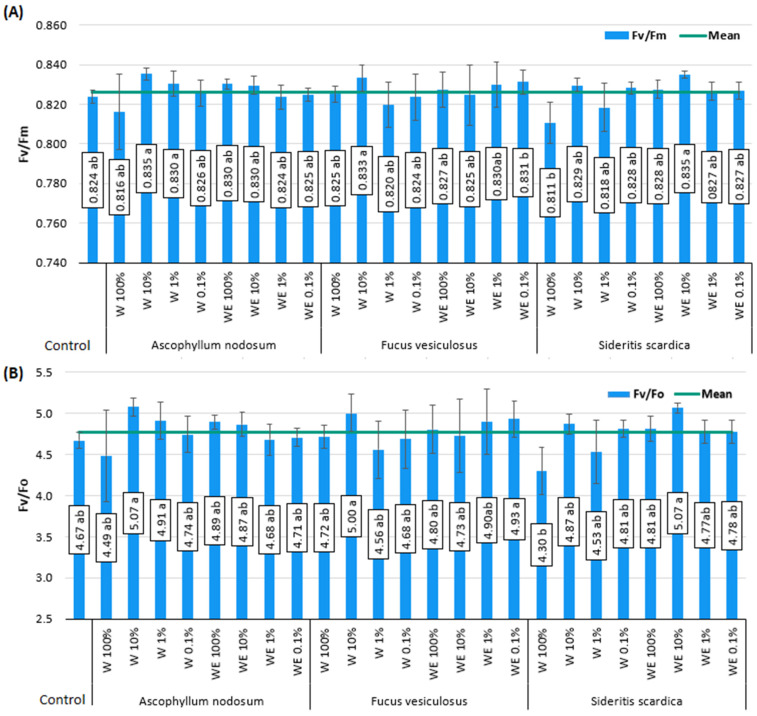

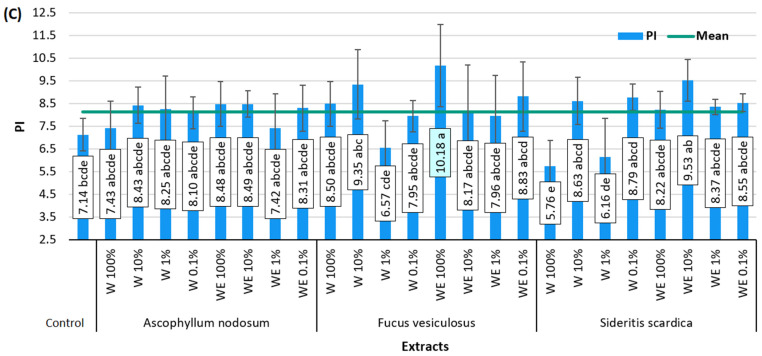
Chlorophyll a fluorescence parameters measured in mung bean microgreens: (**A**) Fv/Fm, (**B**) Fv/Fo, (**C**) PI. W—aqueous extract; WE—hydroethanolic extract. Fv/Fm—Maximal photochemical efficiency of PSII; Fv/Fo—The efficiency of the oxygen evolving complex (OEC) on the donor side of PSII; PI—Performance Index. Values are presented as mean ± SD. Means with the same letters do not differ significantly at *p* < 0.05 in Tukey’s test. The blue label indicates the mean value significantly different from the control.

Figure 3A presents measurements of the Fv/Fm parameter, representing the maximum quantum efficiency of PSII. Values across all tested treatments ranged from 0.81 to 0.835. The differences were minimal, with most values not differing significantly from the control, indicating that young leaves remained in good physiological condition under all treatments.

Figure 3B shows the Fv/Fo parameter, which reflects the efficiency of the oxygen evolving complex (OEC) on the donor side of PSII. Overall, the extracts maintained primary photochemical efficiency at levels comparable to the control, with the exception of the 0.1% hydroethanolic extract of *Fucus vesiculosus*, which slightly reduced the Fv/Fo value.

Figure 3C illustrates the Performance Index (PI), reflecting the efficiency of light energy conversion into chemical energy. Higher PI values correspond to better photosynthetic performance. This index is highly sensitive to environmental changes, often before visible symptoms occurs. The control plants had a PI of 7.14. Slightly elevated PI values were observed for 10% hydroethanolic extract of *A. nodosum* (8.49) and 0.1% hydroethanolic extract of *S. scardica* (8.55). The lowest values were observed for 1% hydroethanolic extract of *F. vesiculosus* (6.57) and 100% aqueous extract of *S. scardica* (5.76).

To summarize Figure 3, the Fv/Fm ratio remained stable, with no significant differences between the variants, suggesting that photosystem II remained in good condition regardless of the extract used. All values were within the range of 0.81–0.835, indicating that photosystem II functioned properly and no photoinhibitory stress occurred. The photosynthetic efficiency index (PI) appeared to be more responsive to the applied treatments than the remaining fluorescence parameters. Slightly higher PI values were observed for the 100% hydroethanolic extract of *F. vesiculosus*.

### 2.6. Total Phenolic and Flavonoid Content

**Figure 4:**
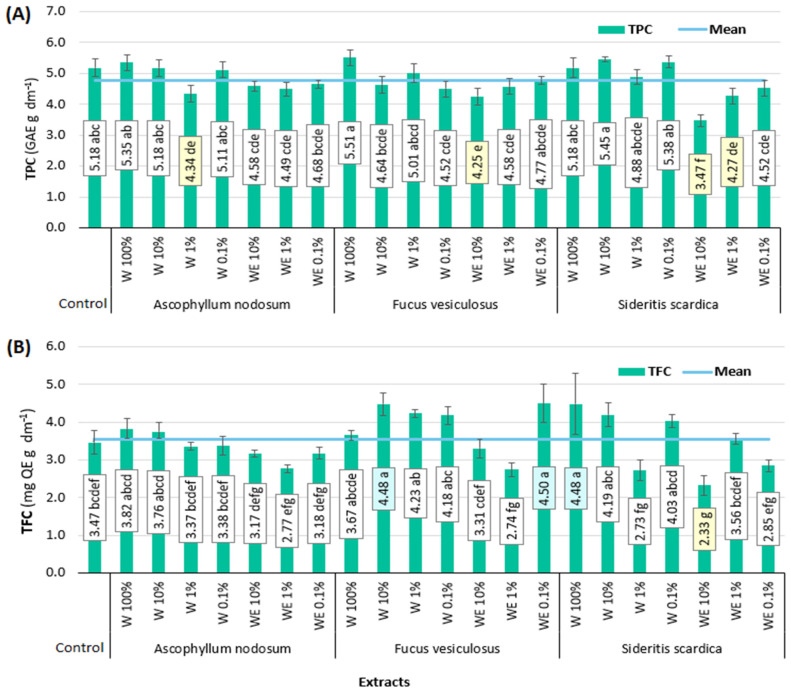
Total phenolic content of (TPC; (**A**)) and total flavonoid content (TFC; (**B**)) of in mung bean microgreens as affected by extract type and concentration. W—aqueous extract; WE—hydroethanolic extract. GAE—gallic acid equivalent, QE—quercetin. Mean values ± SD. Means with the same letters do not differ significantly at *p* < 0.05 in Tukey’s test. Blue labels indicate mean values higher than the control, whereas yellow labels indicate mean values lower than the control.

Figure 4A illustrates the effect of the extracts on the total phenolic content (TPC) in young mung bean plants. Lower TPC values relative with the control were observed for plants treated with 1% aqueous extract of *A. nodosum*, 10% hydroethanolic extract of *F. vesiculosus*, and 1% and 10% hydroethanolic extracts of *S. scardica*.

Regarding total flavonoid content (TFC) (Figure 4B), this parameter was most strongly affected by extracts of *Sideritis scardica*.

**Figure 5:**
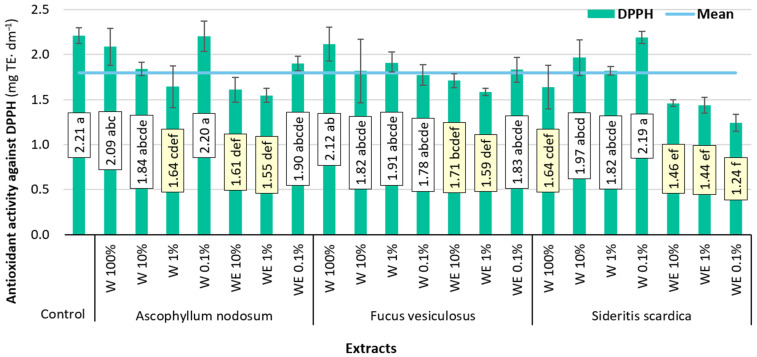
Antioxidant activity determined using the DPPH assay in mung bean microgreens as affected by extract type and concentration. TE—Trolox equivalent antioxidant capacity. Values are presented as mean ± SD. Means with the same letters do not differ significantly at *p* ≤ 0.05 according to Tukey’s test. Yellow labels indicate mean values lower than the control.

The application of hydroethanolic extracts resulted in a decrease in antioxidant activity (Figure 5). Particularly pronounced effects were observed after the application of hydroethanolic extracts of *S. scardica*.

## 3. Discussion

Germination results indicate that high concentrations of ethanolic extracts (10%) may exert phytotoxic effects, significantly reducing germination energy and capacity. In contrast, aqueous and hydroethanolic extracts maintained germination parameters at levels comparable to the control. Similar observations were reported by Drygaś et al. [25], who found that low concentrations of aqueous algal extracts did not impair kale germination parameters. Silva et al. [28] also demonstrated that hydroethanolic preparations of the brown algae *Ascophyllum nodosum* and *Sargassum muticum* affected germination responses in rice and lettuce in a concentration-dependent manner at concentrations ranging from 0 to 100%. The highest germination percentage and Maguire index values were recorded for the 25% extracts, whereas the lowest values were observed for the 100% treatments. These findings are consistent with our observations, in which low concentrations of aqueous algal extracts did not negatively affect mung bean germination. Salehi et al. [29] investigated the effects of ethanol on the germination of three turfgrass species. Germination rates increased at low ethanol concentrations (up to 0.5% in *Lolium perenne* L. and 0.3% in *Festuca arundinacea* Schreb.), whereas higher concentrations reduced germination. Exposure to 10% ethanol completely inhibited seed germination in both species. In contrast, *Cynodon dactylon* L. showed increased germination at the 3% ethanol concentration. Similarly, Drygaś et al. [25] reported that all ethanol-based extracts negatively affected germination parameters. Aulya [30] evaluated the effects of aqueous and ethanolic extracts of *Acacia nilotica* leaves on mung bean (*Vigna radiata*) germination. Lower extract concentrations accelerated germination, increased radicle length, and improved seedlings fresh weight, whereas no negative effects on germination were observed. The authors suggested that *A. nilotica* extracts may act as natural biostimulants with potential applications in agriculture and microgreen production [30].

Miyoshi and Sato [31] investigated the effects of ethanol on germination of Japanese and Indian rice (*Oryza sativa* L.) under aerobic and anaerobic conditions. In dehulled Japonica rice seeds, low ethanol concentrations (0.5–5%) alleviated the inhibitory effect of dehulling and promoted germination, whereas higher concentrations suppressed germination. In intact seeds, ethanol generally inhibited germination or had little effect. Under anaerobic conditions, ethanol counteracted the germination-promoting effect of oxygen deprivation.

In Indian rice, low ethanol concentrations slightly suppressed germination, whereas 2–3% ethanol promoted germination as seed maturity advanced. However, concentrations of 5–6% completely inhibited germination. Similar to Japonica rice, ethanol in unhulled seeds either inhibited germination or showed minimal stimulatory effects, whereas its effects under anaerobic conditions were negligible.

In the study by El-Mahy [32], the effects of ethanolic extracts from Euphorbia prostrata on germination and growth of several plant species were evaluated. The response depended on both extract concentration and plant species. Lower extract concentrations (0.4–2 ppm) had little effect on germination, whereas higher concentrations (4 ppm) significantly inhibited germination, particularly in carrots, tomatoes, lettuce, and onions. The inhibitory effect was more pronounced for root than for shoot growth. The authors suggested that the extract may have allelopathic potential depending on concentration and plant species [32].

Similarly, ethanolic extracts from *Leucaena leucocephala* leaves were reported to contain allelopathic compounds, including flavonoids and phenolic compounds, capable of affecting plant growth and germination [33]. Higher extract concentrations (20 mg∙mL^−1^) significantly reduced germination, seedling growth, biomass accumulation, chlorophyll content, and water uptake.

In the kale experiment conducted by Drygaś et al. [25], higher concentrations of hydroethanolic extracts negatively affected microgreens growth and fresh weight, particularly at the 10% concentration. However, these inhibitory effects were less pronounced in mung bean, suggesting greater tolerance of this species to unfavorable extract treatments.

The inhibitory effects observed at higher concentrations of ethanolic and hydroethanolic extracts may be associated with the higher extraction efficiency of ethanol toward phenolic compounds and other secondary metabolites. Phenolic acids and flavonoids can exhibit allelopathic activity, thereby affecting membrane permeability, mitochondrial respiration, and cellular metabolism in plants [34,35]. These compounds may also disrupt membrane integrity, impair ion transport and energy metabolism [36]. In addition, they may inhibit the mobilization of seed storage reserves through reduced hydrolytic enzyme activity, including α-amylase [37], and alter phytohormone balance, particularly gibberellins and abscisic acid [35].

The changes observed in TPC, TFC, and antioxidant activity in mung bean microgreens may be associated with bioactive compounds present in the applied *Sideritis scardica* extracts (Table 1). Phenolic acids, phenylethanoid glycosides, and flavonoids are known to act as signaling molecules involved in regulating plant metabolism and stress responses, including the phenylpropanoid pathway [38]. However, the decrease in antioxidant activity observed in the present study may suggest reduced oxidative stress in plant tissues. Under lower oxidative pressure, plants often synthesize smaller amounts of endogenous antioxidant compounds due to a reduced demand for radical scavenging molecules [39]. Similar responses have been reported following biostimulant application, where improved physiological status was associated with changes in antioxidant metabolism [40]. Therefore, the reduced DPPH radical scavenging activity observed in mung bean microgreens may reflect improved redox balance rather than a negative physiological response. In this context, phenolic compounds identified in *S. scardica* extracts may act as metabolic regulators, influencing the synthesis of endogenous phenolics and flavonoids in plant tissues. Such responses are consistent with the proposed biostimulant potential of plant- and algae-derived extracts.

The experimental results demonstrate that the application of 10% hydroethanolic extracts reduced total phenolic content and antioxidant activity (DPPH) in mung bean plants. In the study by Drygaś et al. [25], no comparable negative effects of the tested extracts on kale microgreens were observed, although a tendency toward reduced DPPH scavenging activity was also reported for concentrated hydroethanolic extracts.

The stability of chlorophyll a fluorescence parameters indicates that the extracts did not induce photoinhibitory stress and did not adversely affect plant physiological status.

The elevated content of phenolic compounds and flavonoids observed in plants treated with *Fucus vesiculosus* extracts suggests the potential of this alga as a source of bioactive secondary metabolites [41,42,43,44]. The relatively stable chlorophyll fluorescence parameters, particularly Fv/Fm and PI, suggest that the applied extracts did not severely affect PSII functioning despite changes in antioxidant activity and phenolic metabolism. This may indicate that the physiological responses induced by the extracts occurred primarily at the metabolic level without substantial impairment of the photosynthetic apparatus. Together with the observed growth responses, these results suggest that the ex-tracts influenced plant physiology mainly through metabolic regulation rather than direct impairment of photosynthetic efficiency.

The slight reduction in chlorophyll content observed after exposure to the highest concentration of the hydroethanolic extract of *S. scardica* may be related to the high levels of phenolic compounds identified in this extract (Table 1), including caffeoylquinic acid derivatives, phenylethanoid glycosides (e.g., verbascoside and echinacoside), and flavone glycosides (e.g., hypolaetin and isoscutellarein). These compounds are known to influence plant redox balance and photosynthetic metabolism, and their effects are often concentration-dependent [38,39]. In contrast, these compounds were present at lower concentrations in the aqueous extract. The observed changes in phenolic and flavonoid content, together with differences in antioxidant activity, may therefore indicate alterations in the oxidative balance of mung bean plants. Similar physiological responses have been reported in plants exposed to allelochemicals, where secondary metabolites affect antioxidant metabolism and stress-related pathways [45,46,47].

Ethanolic and hydroethanolic extracts of *S. scardica* contain various bioactive compounds, including rosmarinic acid, verbascoside, flavonoids, tannins, and terpenes, which may induce oxidative and physiological responses in plant tissues [48,49,50,51,52]. Previous studies have shown that such compounds can stimulate antioxidant defense mechanisms and affect oxidative balance [49,53,54]. These responses may follow a biphasic, hormetic pattern, in which low extract concentrations promote adaptive responses, whereas high concentrations may lead to ROS accumulation, lipid peroxidation, and growth inhibition [47,53,54]. This suggests that the effects of *S. scardica* extracts on mung bean plants may depend on both extract concentration and chemical composition.

The differences observed in TPC and TFC in mung bean microgreens (Figure 4) may also be partially related to the phlorotannin composition of the algal extracts (Table 4). Phlorotannins such as phloroglucinol, phlorethol, fucophloretol, and fuhalol were detected exclusively in aqueous extracts and were not detected in hydroethanolic extracts. This may explain why aqueous extracts generally resulted in higher TPC and TFC values in microgreens. Moreover, the total phlorotannin content was substantially higher in F. vesiculosus (8.28 µg mL^−1^) than in *A. nodosum* (2.76 µg mL^−1^), which may have contributed to the greater accumulation of phenolic compounds and flavonoids observed in plants treated with *F. vesiculosus* extracts.

The analysis of chlorophyll fluorescence parameters (Fv/Fm, Fv/Fo, and PI) in mung bean sprouts provides insight into the physiological responses associated with the applied extracts. The absence of major differences between the control and treated plants suggests that photosystem II (PSII) was not substantially affected. These parameters are commonly used to evaluate photochemical stress and photoinhibition, and their stability suggests that PSII remained functional [55].

Previous studies have shown that phenolic compounds and related metabolites may induce the formation of reactive oxygen species (ROS) and activate plant defense mechanisms before visible damage to PSII occurs [53,56]. In the present study, the increase in total phenolic and flavonoid content together with stable chlorophyll fluorescence parameters may indicate a mild stress-related adaptive response in mung bean plants. Similar responses have been described as hormetic effects, where low or moderate stress levels stimulate antioxidant metabolism and the accumulation of phenolic compounds, whereas higher doses may result in growth inhibition or oxidative stress [57,58,59,60,61,62,63].

Overall, the physiological responses observed in this study may result from interactions between bioactive metabolites present in the extracts and plant metabolic processes. These interactions may involve ROS signaling, modulation of phenylpropanoid metabolism, and hormonal regulation, which are commonly associated with the biostimulant effects of seaweed-derived preparations and may explain the concentration-dependent responses observed in this study.

## 4. Materials and Methods

The study was carried out in two stages. In the first phase of the study, the effects of the extracts on seed germination, germination energy, and germination capacity was evaluated. This assessment was performed by monitoring seed germination in Petri dishes. In the subsequent stage, the influence of the tested extracts on the early growth and chemical composition of plants cultivated on linen mats was assessed.

### 4.1. Preparation of Extracts

Aqueous extracts were prepared in accordance with the methodology described by Drygaś et al. [15,64], while hydroethanolic and ethanolic extracts were prepared according to the methods described by Drygaś et al. [25]. A comprehensive analysis of the extracts composition is provided in the aforementioned study [25]. All extracts were prepared from commercially available dried plant and seaweed materials. *Fucus vesiculosus* (fragmented algal material including bladders) was purchased from Dary Podlasia Adam Nowicki (Bielsk Podlaski, Poland), *Sideritis scardica* (whole dried plants) from RAFEX (Ciecierzyn, Poland), and *Ascophyllum nodosum* (brown algal powder) from RAFEX (Ciecierzyn, Poland). The raw materials were stored in dry conditions at room temperature until extraction.

The undiluted extracts were designated as the 100% extract. Subsequently, serial dilutions (10%, 1%, 0.1%, and 0.01%) were prepared using distilled water. Ethanol was not evaporated or removed after extraction, and the extracts were used directly for subsequent analyses. The resulting treatment variants are presented in Table 7.

### 4.2. Determination of Polyphenolic Compounds Profile in Extracts

Determination of polyphenolic compounds was carried out using the Ultra Performance Liquid Chromatography (UPLC) Waters ACQUITY system (Waters, Milford, MA, USA). UPLC was equipped with a binary pump manager, column manager, sample manager, photodiode array (PDA) detector, and tandem quadrupole mass spectrometer (TQD) with an electrospray ionization (ESI) source. Separation of polyphenolic compounds was performed using a 1.7 µm, 100 mm × 2.1 mm UPLC BEH RP C18 column (Waters, Milford, MA, USA). The mobile phase consisted of 0.1% formic acid in water, *v*/*v* (solvent A) and 0.1% formic acid in 40% acetonitrile, *v*/*v* (solvent B). The flow rate was kept constant at 0.35 mL/min for a total run time of 8 min. The system was run using the following gradient program: from 0 min 5% B, from 0 to 8 min, a linear to 100% B, and from 8 to 9.5 min for washing and back to initial conditions. The injection volume of the samples was 5 µL, and the column was maintained at 50 °C. The following TQD parameters were used: cone voltage of 30 V, capillary voltage of 3500 V, source and desolvation temperatures of 120 °C and 350 °C, respectively, and desolvation gas flow rate of 800 L/h.

Characterization of the individual polyphenolic compounds was performed on the basis of retention times, mass-to-charge ratios, and fragment ions, using commercial standards of 3-O-caffeoylquinic acid, *p*-coumaric acid, verbascoside and phloroglucinol, as well as literature data. The obtained data were processed in Waters MassLynx v.4.1 software (Waters, Milford, MA, USA). The analyses were performed using the most concentrated extracts, designated as 100%. The analyses of the extracts were carried out in triplicate.

### 4.3. Germination Test

The experimental material consisted of mung bean (*Vigna radiata*) seeds (TORAF, Maciejów, Poland, batch number 86928CIN0ES) certified for organic farming under EU standards. The initial experiment was conducted in a growth chamber at 20 °C in darkness.

The experiment was conducted for 30 days, while germination parameters were assessed during the initial phase of seed development.

Germinating seeds were counted daily. The germination test was performed on 8.5 cm Petri dishes, lined with six layers of medium-porosity filter paper, with three replicates for each treatment. Each filter paper was moistened with 5 mL of the corresponding extract, and 25 seeds were sown per dish. The extracts were applied once using an automatic pipette.

### 4.4. Pot Experiment

#### 4.4.1. Experimental Conditions

The experiment was conducted using a completely randomized design with three replicates per treatment and focused on mung bean cultivation in container systems. The growth substrate comprised 200 cm^2^ flax fiber mats (5 mm thickness; T-shop, Wrocław, Poland) used for cultivating microgreens under controlled conditions. Each mat was thoroughly saturated with 50 mL of the respective extract, and seeds were sown at a density of 12 g per container.

The study was conducted within a growth chamber maintained at 20 °C in darkness until the onset of seed germination. After germination, the containers were transferred to an conditions promoting to photomorphogenesis. The chamber provided photosynthetically active radiation (PAR) within the 400–700 nanometer range, with a photoperiod of 10 h of light and 14 h of darkness. On the third day, the germinating seeds were re-irrigated with 50 mL of extract per mat, resulting in a total extract volume of 100 mL of extract per container. The plants were subsequently watered with distilled water.

On the thirteenth day plants were subsequently harvested simultaneously across all treatment groups. This harvest occurred by cutting the plants just above the mat surface. Following this harvest, the plants were subjected to further analyses. At the time of harvest, plants exhibited fully developed cotyledons and the first true leaves.

#### 4.4.2. Measurement of Selected Parameters Related to Photosynthesis

Before harvesting plants, physiological measurements of relative chlorophyll content and selected chlorophyll fluorescence parameters were conducted. All measurements were performed on fully expanded leaves under controlled conditions, at an ambient temperature of approximately 22 °C.

##### Relative Chlorophyll Content Measurement

Relative chlorophyll content was determined using a handheld CCM-200plus Chlorophyll Content Meter (Opti-Sciences, Hudson, NH, USA), which measures the absorbance of light by chlorophyll a and b at wavelengths of 650 nm and 940 nm. Measurements were expressed as a relative unit, CCI (chlorophyll content index). Measurements were taken on twenty leaves per each container.

##### Chlorophyll a Fluorescence Measurement

Chlorophyll a fluorescence parameters (Fv/Fm, Fv/Fo, and PI) were determined using a Pocket PEA fluorometer (Hansatech Instruments, King’s Lynn, Norfolk, UK). Before measurement, the leaves were dark-adapted for 30 min using PEA clips to ensure full opening of all photosystem II (PSII) reaction centers. Following dark adaptation, the induction of chlorophyll fluorescence (Kautsky curve) was recorded in response to a saturating red light pulse (approx. 3500 µmol m^−2^ s^−1^) applied for 1 s. The following parameters were analyzed: the efficiency of the oxygen evolving complex (OEC) on the donor side of PSIII (Fv/Fo), the maximum quantum yield of PSII photochemistry (Fv/Fm), and the performance index of PSII (PI) [65,66,67]. In each experimental treatment, chlorophyll fluorescence measurements were taken on six plants (two measurements per container).

### 4.5. Assays for Antioxidant Activity

Samples used for the determination of antioxidant activity, total phenolic content (TPC), and total flavonoid content (TFC) were prepared according to the procedure described by Drygaś et al. [25]. Antioxidant activity was evaluated using DPPH radicals, and the results were expressed as Trolox equivalents (mg TE g^−1^ dry weight). TPC results were expressed as gallic acid equivalents (mg GAE g^−1^ dry weight), while TFC results were expressed as quercetin equivalents (mg QE g^−1^). Analyses of TPC, TFC, and DPPH radical scavenging activity in microgreens were performed in three replicates.

### 4.6. Statistical Analysis

Statistical analyses were carried out using analysis of variance (ANOVA) followed by Tukey’s post hoc test, with significance set at *p* < 0.05. All analyses were performed using Statistica software version 13.3.0 (TIBCO Software Inc., Palo Alto, CA, USA).

## 5. Conclusions

The study demonstrated that the effect of plant and algal extracts on mung bean germination depended strongly on the extraction solvent and extract concentration. Aqueous and hydroethanolic extracts generally did not adversely affect germination parameters, maintaining germination energy and capacity at high levels (96–100%), comparable to the control. In contrast, the application of 10% ethanolic extracts significantly inhibited germination. The strongest inhibitory effect was observed for the ethanolic extract of *Sideritis scardica*, where germination energy decreased to 8% and germination capacity reached only 42.7–46.7%. Similarly, ethanolic extracts of *Ascophyllum nodosum* and *Fucus vesiculosus* reduced germination energy to 24% and 40%, respectively, and final germination capacity to 57% and 60%. The Pieper index further confirmed the negative effects of concentrated ethanolic extracts.

Extract application also influenced biochemical parameters in mung bean microgreens, including total phenolic content (TPC), total flavonoid content (TFC), and antioxidant activity. Chromatographic analysis revealed numerous phenolic compounds in *S. scardica* extracts, while phlorotannins were identified in algal extracts, with higher total contents in *F. vesiculosus* than in *A. nodosum*.

Overall, the results indicate that aqueous and low-concentration hydroethanolic extracts can be applied without negatively affecting mung bean germination or early physiological status, whereas concentrated ethanolic extracts showed inhibitory effects under the tested conditions. These findings highlight the importance of solvent type and extract concentration in determining the biological activity of plant- and algae-derived extracts and may contribute to the development of optimized plant- and algae-based biostimulant formulations.

## Figures and Tables

**Table 1:** Individual phenolic compounds identified by UPLC-PDA-MS/MS in *Sideritis scardica* extract.

Compound		Rt	λ_max_	[M − H] *m*/*z*	
		min	nm	MS	MS/MS
	*Tannins*				
1	3-*O*-caffeoylquinic acid	3.19	324	353	191
2	*p*-Coumaroylmelittoside	3.99	312	669	163
3	Hypolaetin 7-*O*-glucoside-(1→2)-glucoside	5.17	276, 338	625	301
4	Echinacoside	5.44	329	785	623, 179, 135
5	Forsythoside B/Samioside	5.55	329	755	593, 179, 161, 135
6	Verbascoside	5.69	329	623	461, 179, 161, 135
7	Hypolaetin 7-*O*-glucoside-(1→2)-rhamnoside	5.78	276, 338	609	301
8	Forsythoside B/Samioside	5.96	331	755	593, 179, 161, 135
9	Hypolaetin 7-*O*-(6′-acetyl)glucoside(1→2)-glucoside	6.14	276, 338	667	625, 301
10	4′-Methylhypolaetin 7-*O*-glucoside-(1→2)-glucoside	6.21	276, 338	639	301
11	Alyssonoside	6.41	329	769	593, 461, 175
12	LeucoseptosideA	6.61	329	637	461, 193
13	Isoscutellarein 7-*O*-(6′-acetyl)hexoside(1→2)-hexoside isomer I	6.69	276, 338	651	285
14	Isoscutellarein 7-*O*-(6′-acetyl)hexoside(1→2)-hexoside isomer II	6.89	276, 338	651	285
15	4′-Methylhypolaetin 7-O-(6′-acetyl)glucoside(1→2)-glucoside	7.24	276, 338	681	639, 301
16	Leontoside B	7.51	328	783	193, 175
17	4′-Methylisoscutellarein 7-*O*-(6′-acetyl)hexosyl(1→2)-[6′-acetyl]-glucoside	8.14	274, 338	707	301
18	Isoscutellarein 7-O-(6′-acetyl)hexosyl(1→2)-[6′-acetyl]-hexoside isomer I	8.92	276, 338	693	285
19	Isoscutellarein 7-O-(6′-acetyl)hexosyl(1→2)-[6′-acetyl]-hexoside isomer II	8.99	276, 338	693	285
20	4′-Methylhypolaetin 7-O-(6′-acetyl)hexosyl(1→2)-[6′-acetyl]-hexoside	9.22	276, 338	723	301

**Table 2:** Interaction of compound and extract type.

No.	Type of Compound (C)	Amount of Compound (μg/mL)		
		Type of Extract (E)		
		Aqueous	Hydroethanolic	Ethanolic
1	3-O-caffeoylquinic acid	1.92 ± 0.03 c	7.73 ± 0.10 a	4.66 ± 0.15 b
2	p-Coumaroylmelittoside	0.16 ± 0.00 c	0.85 ± 0.03 a	0.56 ± 0.01 b
3	Hypolaetin 7-O-glucoside-(1→2)-glucoside	0.40 ± 0.01 c	1.13 ± 0.02 a	0.55 ± 0.00 b
4	Echinacoside	0.54 ± 0.01 c	1.37 ± 0.01 b	1.54 ± 0.01 a
5	Forsythoside B/Samioside	9.62 ± 0.09 c	16.08 ± 0.12 b	17.84 ± 0.63 a
6	Verbascoside	7.26 ± 0.05 c	12.89 ± 0.46 b	12.71 ± 0.18 b
7	Hypolaetin 7-O-glucoside-(1→2)-rhamnoside	2.10 ± 0.07 c	7.69 ± 0.11 a	2.97 ± 0.10 b
8	Forsythoside B/Samioside	1.50 ± 0.02 c	3.46 ± 0.12 a	2.39 ± 0.01 b
9	Hypolaetin 7-O-(6′-acetyl)glucoside(1→2)-glucoside	2.23 ± 0.08 c	3.73 ± 0.01 a	3.42 ± 0.09 b
10	4′-Methylhypolaetin 7-O-glucoside-(1→2)-glucoside	1.37 ± 0.01 c	4.81 ± 0.13 a	1.94 ± 0.03 b
11	Alyssonoside	0.99 ± 0.03 c	2.09 ± 0.04 a	2.15 ± 0.10 b
12	LeucoseptosideA	0.51 ± 0.01 c	0.94 ± 0.04 a	0.89 ± 0.00 b
13	Isoscutellarein 7-O-(6′-acetyl)hexoside(1→2)-hexoside isomer I	0.34 ± 0.02 c	0.53 ± 0.00 a	0.39 ± 0.01 b
14	Isoscutellarein 7-O-(6′-acetyl)hexoside(1→2)-hexoside isomer II	11.28 ± 0.02 c	23.22 ± 0.81 a	17.61 ± 0.17 b
15	4′-Methylhypolaetin 7-O-(6′-acetyl)glucoside(1→2)-glucoside	8.53 ± 0.30 c	17.03 ± 0.16 a	12.92 ± 0.89 b
16	Leontoside B	0.31 ± 0.00 c	0.78 ± 0.05 a	0.54 ± 0.01 b
17	4′-Methylisoscutellarein 7-O-(6′-acetyl)hexosyl(1→2)-[6′-acetyl]-glucoside	0.44 ± 0.03 c	0.73 ± 0.01 a	0.65 ± 0.02 b
18	Isoscutellarein 7-O-(6′-acetyl)hexosyl(1→2)-[6′-acetyl]-hexoside isomer I	2.00 ± 0.02 c	3.56 ± 0.10 a	2.93 ± 0.04 b
19	Isoscutellarein 7-O-(6′-acetyl)hexosyl(1→2)-[6′-acetyl]-hexoside isomer II	0.49 ± 0.01 c	1.20 ± 0.02 a	0.83 ± 0.01 b
20	4′-Methylhypolaetin 7-O-(6′-acetyl)hexosyl(1→2)-[6′-acetyl]-hexoside	2.11 ± 0.03 c	3.85 ± 0.03 a	3.28 ± 0.12 b
Mean		2.71 ± 3.41	5.68 ± 6.47	4.54 ± 5.67
C		F = 4023.17; *p* < 0.0001		
E		F = 2235.88; *p* < 0.0001		
C × E		F = 163.63; *p* < 0.0001		

Values followed by different letters within a row differ significantly according to Tukey’s test (*p* < 0.05).

**Table 3:** Individual phlorotannins identified by UPLC-PDA-MS/MS.

Compound	Rt	λ_max_	[M − H] *m*/*z*	
	min	nm	MS	MS/MS
Phloroglucinol	1.76	267	125^-^	109
Phlorethol	1.85	264	249^-^	126
Fucophloretol	1.89	266	374^-^	265
Fuhalol	1.92	269	265^-^	142

**Table 4:** Individual phlorotannins identified by UPLC-PDA MS/MS depending on the type of material and solvent used.

Compound µg/mL	Type of Extract					
	*Ascophyllum nodosum*			*Fucus vesiculosus*		
	Aqueous (W)	Hydroethanolic (W-E)	Ethanolic	Aqueous (W)	Hydroethanolic (W-E)	Ethanolic
Phloroglucinol	1.10 ± 0.01	-	-	2.99 ± 0.07	-	-
Phlorethol	0.24 ± 0.0	-	-	0.92 ± 0.00	-	-
Fucophloretol	-	-	-	0.40 ± 0.00	-	-
Fuhalol	1.42 ± 0.05	-	-	3.97 ± 0.04	-	-
Total	2.76 ± 0.04	-	-	8.28 ± 0.03	-	-

**Table 5:** Summary of the effects of different extract types and concentrations on mung bean germination.

Extract Type	Concentration	Germination Energy %	Germination Capacity After 7 Days (%)	Main Effect
Aqueous	all concentrations	96–100	96–100	no inhibitory effect
Hydroethanolic	≤1%	97–100	99–100	no inhibitory effect
Hydroethanolic	10%	93–97	93–97	slight reduction
Ethanolic	≤1%	99–100	99–100	no inhibitory effect
Ethanolic	10%	8–40	43–60	strong inhibition

**Table 6:** Length and fresh weight of mung bean microgreens depending on extract type and concentration.

Extract		Microgreens Length (cm)	Microgreens Fresh Weight (g)
Control		13.2 ± 1.4 abcd	0.305 ± 0.034 abcde
*Ascophyllum nodosum*	W 100%	10.1 ± 2.4 ef	0.284 ± 0.031 cde
	W 10%	13.2 ± 2.6 abcd	0.313 ± 0.053 abcde
	W 1%	12.6 ± 1.2 bcd	0.308 ± 0.007 abcde
	W 0.1%	13.0 ± 1.4 abcd	0.311 ± 0.010 abcde
	WE 10%	8.1 ± 2.6 fg	0.268 ± 0.020 de
	WE 1%	11.9 ± 1.4 de	0.298 ± 0.009 bcde
	WE 0.1%	12.9 ± 1.4 abcd	0.303 ± 0.012 abcde
*Fucus vesiculosus*	W 100%	10.5 ± 1.4 e	0.294 ± 0.008 bcde
	W 10%	12.8 ± 2.3 abcd	0.323 ± 0.013 abcd
	W 1%	13.4 ± 1.4 abcd	0.349 ± 0.018 ab
	W 0.1%	13.6 ± 1.6 abcd	0.316 ± 0.009 abcde
	WE 10%	8.0 ± 2.8 g	0.263 ± 0.007 e
	WE 1%	12.0 ± 1.8 cde	0.297 ± 0.003 bcde
	WE 0.1%	13.8 ± 0.9 abcd	0.312 ± 0.009 abcde
*Sideritis scardica*	W 100%	13.3 ± 1.5 abcd	0.357 ± 0.014 a
	W 10%	14.0 ± 1.7 abc	0.331 ± 0.014 abc
	W 1%	14.7 ± 1.9 a	0.329 ± 0.013 abc
	W 0.1%	14.1 ± 1.5 ab	0.334 ± 0.014 abc
	WE 10%	8.0 ± 2.6 g	0.289 ± 0.005 cde
	WE 1%	14.6 ± 1.3 ab	0.332 ± 0.009 abc
	WE 0.1%	14.3 ± 0.9 ab	0.339 ± 0.013 abc

Values are presented as mean ± SD. W—aqueous extract; WE—hydroethanolic extract. Means within columns marked with the same letters do not differ significantly at *p* < 0.05 in Tukey’s test.

**Table 7:** Experimental variants of extracts according to source material, extract concentration, and extraction solvent.

Symbol	Solvent/Concentration	Description
Control	–	distilled water only
*Ascophyllum nodosum*	W: 100%, 10%, 1%, 0.1%, 0.01%	aqueous extracts (undiluted to 0.01%)
	WE: 10%, 1%, 0.1%, 0.01%	hydroethanolic extracts (10% to 0.01%)
	E: 10%, 1%, 0.1%, 0.01%	ethanolic extracts (1% to 0.01%)
*Fucus vesiculosus*	W: 100%, 10%, 1%, 0.1%, 0.01%	aqueous extracts (undiluted to 0.01%)
	WE: 10%, 1%, 0.1%, 0.01%	hydroethanolic extracts (10% to 0.01%)
	E: 10%, 1%, 0.1%, 0.01%	ethanolic extracts (1% to 0.01%)
*Sideritis scardica*	W: 100%, 10%, 1%, 0.1%, 0.01%	aqueous extracts (undiluted to 0.01%)
	WE: 10%, 1%, 0.1%, 0.01%	hydroethanolic extracts (10% to 0.01%)
	E: 10%, 1%, 0.1%, 0.01%	ethanolic extracts (10% to 0.01%)

## Data Availability

The original contributions presented in this study are included in the article/Supplementary Material. Further inquiries can be directed to the corresponding author.

## Supplementary Materials

The following supporting information can be downloaded at: https://www.mdpi.com/article/10.3390/molecules31122030/s1, Table S1 Germination energy and germination capacity of mung bean seeds treated with different extract types and concentrations; Figure S1 Parameters describing Mung bean germination as a function of the type and extract concentration.
