# Supplementary material for: Biostimulatory and Inhibitory Effects of Natural Extracts on Vigna radiata: Concentration-Dependent Responses
Source: Molecules. 2026 Jun 10;31(12):2030. doi: 10.3390/molecules31122030 (PMC13304841; doi:10.3390/molecules31122030)
Supplement: Supplementary file 1 [file molecules-31-02030-s001.zip › molecules-4290940-supplementary.pdf]

Table S1. Germination energy and germination capacity of mung bean seeds treated with different extract types and concentrations

Extract		Germination Energy (%) after 5 days	Germination Capacity (%)	
			after 7 days	after 12 days
Control		99.5 ± 1.4 a	99.5 ± 1.4 a	99.5 ± 1.4 a
Ascophyllum nodosum	W 100%	100.0 ± 0.0 a	100.0 ± 0.0 a	100.0 ± 0.0 a
	W 10%	100.0 ± 0.0 a	100.0 ± 0.0 a	100.0 ± 0.0 a
	W 1%	96.0 ± 4.0 a	96.0 ± 4.0 a	96.0 ± 4.0 a
	W 0.1%	100.0 ± 0.0 a	100.0 ± 0.0 a	100.0 ± 0.0 a
	W 0.01%	100.0 ± 0.0 a	100.0 ± 0.0 a	100.0 ± 0.0 a
	WE 10%	93.3 ± 11.5 a	93.3 ± 11.5 a	93.3 ± 11.5 a
	WE 1%	98.7 ± 2.3 a	98.7 ± 2.3 a	98.7 ± 2.3 a
	WE 0.1%	100.0± 0.0 a	100.0 ± 0.0 a	100.0 ± 0.0 a
	WE 0.01%	100.0 ± 0.0 a	100.0 ± 0.0 a	100.0 ± 0.0 a
	E 10%	24.0 ± 14.0 c	57.3 ± 22.6 b	57.3 ± 22.6 b
	E 1%	98.7 ± 2.3 a	98.7 ± 2.3 a	98.7 ± 2.3 a
	E 0.1%	98.7 ± 2.3 a	100.0 ± 0.0 a	100.0 ± 0.0 a
	E 0.01%	98.7 ± 2.3 a	98.7 ± 2.3 a	98.7 ± 2.3 a
Fucus vesiculosus	W 100%	100.0 ± 0.0 a	100.0 ± 0.0 a	100.0 ± 0.0 a
	W 10%	100.0 ± 0.0 a	100.0 ± 0.0 a	100.0 ± 0.0 a
	W 1%	97.3 ± 2.3 a	97.3 ± 2.3 a	97.3 ± 2.3 a
	W 0.1%	100.0 ± 0.0 a	100.0 ± 0.0 a	100.0 ± 0.0 a
	W 0.01%	100.0 ± 0.0 a	100.0 ± 0.0 a	100.0 ± 0.0 a
	WE 10%	96.0 ± 6.9 a	96.0 ± 6.9 a	96.0 ± 6.9 a
	WE 1%	100.0 ± 0.0 a	100.0 ± 0.0 a	100.0 ± 0.0 a
	WE 0.1%	100.0 ± 0.0 a	100.0 ± 0.0 a	100.0 ± 0.0 a
	WE 0.01%	100.0 ± 0.0 a	100.0 ± 0.0 a	100.0 ± 0.0 a
	E 10%	40.0 ± 10.6 b	60.0 ± 8.0 b	60.0 ± 8.0 b
	E 1%	100.0± 0.0 a	100.0 ± 0.0 a	100.0 ± 0.0 a
	E 0.1%	98.7 ± 2.3 a	98.7 ± 2.3 a	100.0 ± 0.0 a
	E 0.01%	98.7 ± 2.3 a	98.7 ± 2.3 a	98.7 ± 2.3 a
Sideritis scardica	W 100%	100.0 ± 0.0 a	100.0 ± 0.0 a	100.0 ± 0.0 a
	W 10%	98.7 ± 2.3 a	98.7 ± 2.3 a	98.7 ± 2.3 a
	W 1%	100.0± 0.0 a	100.0 ± 0.0 a	100.0 ± 0.0 a
	W 0.1%	100.0 ± 0.0 a	100.0 ± 0.0 a	100.0 ± 0.0 a
	W 0.01%	98.7 ± 2.3 a	98.7 ± 2.3 a	98.7 ± 2.3 a
	WE 10%	97.3 ± 4.6 a	97.3 ± 4.6 a	97.3 ± 4.6 a
	WE 1%	98.7 ± 2.3 a	98.7 ± 2.3 a	98.7 ± 2.3 a
	WE 0.1%	100.0± 0.0 a	100.0 ± 0.0 a	100.0 ± 0.0 a
	WE 0.01%	100.0 ± 0.0 a	100.0 ± 0.0 a	100.0 ± 0.0 a
	E 10%	8.0 ± 0.0 d	42.7 ± 6.1 b	46.7 ± 6.1 b
	E 1%	100.0± 0.0 a	100.0 ± 0.0 a	100.0 ± 0.0 a
	E 0.1%	100.0 ± 0.0 a	100.0 ± 0.0 a	100.0 ± 0.0 a
	E 0.01%	100.0 ± 0.0 a	100.0 ± 0.0 a	100.0 ± 0.0 a

Mean values ± SD. W—water (aqueous) extract; WE—water–ethanol extract; E—ethanol extract. Means in columns marked with the same letters do not differ significantly at $p < 0.05$ in Tukey`s test.

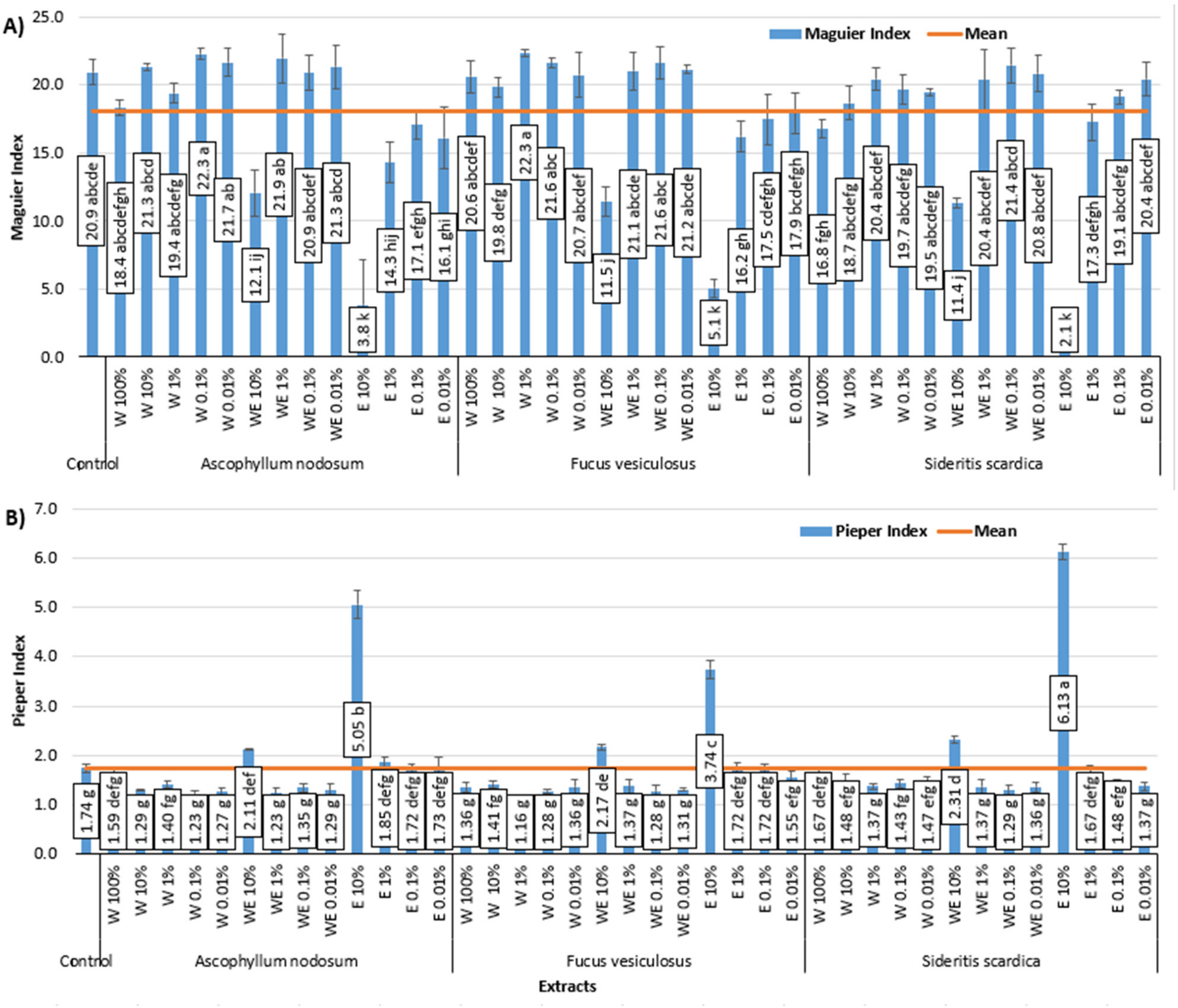

Figure S1. Parameters describing Mung bean germination as a function of the type and dose of the extract. A) Maguier Index; Pieper Index. W—water (aqueous) extract; WE—water–ethanol extract. GAE - gallic acid equivalent, QE – quercetin. Mean values ± SD. Means with the same letters do not differ significantly at $p < 0.05$ in Tukey`s test.
